# The Causes and Treatment of Sterility, with a Preliminary Statement of the Physiology of Generation

**Published:** 1856-09

**Authors:** 


ART. VIII.— *The Causes and Treatment of Sterility*, with a Preliminary Statement of the Physiology of Generation. With colored lithographs and numerous wood-cut illustrations. By AUGUSTUS K. GARDNER, A.M., M. D., etc., etc. New York: DEWITT & DAVENPORT, Publishers. 1856.

This monograph is a well printed octavo of some 170 pages, profusely illustrated. So far as we can judge it makes no pretensions to originality, either in research or treatment. The author considers the causes of sterility as ordinarily recognized, such as imperforate hymen, occluded or diseased os uteri, etc. He avails himself freely of the labors of Tyler Smith and others, and in matters of diagnosis is usually shrewd and satisfactory. The work may be a very useful one, and though we have heard some outcry against it, we do not for ourselves find anything censurable unless it is a stilted, sophomoric tone, which occasionally comes in with ludicrous effect to impair an otherwise excellent discourse.

We have had occasion to say before, that diseases of females, to be described in good taste, require a plain, straightforward, scientific tone of discussion. The author should not reveal a consciousness of being engaged upon a delicate subject, for it is immodest to have any such consciousness. The barren, unornamented, logical style is the only appropriate one; and when we fall upon appeals to the sentiments in such connection, we regard them as very much out of place.

